# Reconfiguring work: artificial intelligence, agentic AI, and the future of the radiology profession

**DOI:** 10.1093/bjrai/ubag006

**Published:** 2026-03-16

**Authors:** Angela Aristidou

**Affiliations:** School of Management, University College London, London, E14 5AA, United Kingdom; UK Research and Innovation Future Leader Fellow; Stanford Digital Economy Lab, Institute for Human-Centered AI, Stanford University, Stanford, California, 94305, United States

**Keywords:** artificial intelligence, future of work, radiology, profession, occupation, organisation

## Abstract

Radiology is undergoing a major shift with the growing use of artificial intelligence (AI), and more change is expected with the emergence of agentic AI—systems that can initiate, manage, and coordinate tasks. So far, most discussions about AI’s impact on radiology follow 2 main approaches. The first, the “displacement” approach, tries to predict which jobs are most at risk of being replaced by AI. This narrative often warns that radiologists may be displaced. The second, the automation-versus-augmentation approach, looks within jobs to identify which tasks are likely to be fully automated (automation) and which will be improved by AI working alongside humans (augmentation). This paper introduces a third approach: **reconfiguration**. Instead of focusing on job loss or task replacement, the reconfiguration model looks at how AI changes the way tasks connect, how responsibilities shift, and how professional roles evolve. Drawing on recent research and developments in AI, this paper advances the reconfiguration approach and articulates why it offers a clearer way to understand—and help shape—the future of work in radiology. This paper offers a forward-looking reflection on the shifting nature of radiological work—clinically, educationally, and organizationally—as AI systems become increasingly integrated into practice.

## Radiology at the forefront of AI: a profession under the microscope

Radiology occupies a distinctive position in the broader story of artificial intelligence, functioning as a testbed for artificial intelligence (AI) capabilities and for debates about the future of professional work—both in healthcare and beyond. As one of the most digitally mature medical specialties, with image data routinely archived in standardized formats,^1^ over a thousand AI tools have now been cleared or approved for tasks ranging from image triage to segmentation,^2^ and radiology is often cited as the domain where AI is likely to achieve the earliest and most visible impact on expert practice [eg,^3^].

Against this backdrop, radiology is viewed in two contrasting yet complementary ways: as the *tip of the spear*, leading AI integration into clinical practice, and as the *canary in the coal mine*, offering early signals of how AI may transform professional work across other healthcare professions and other knowledge-intensive fields of work.

This dual positioning has drawn sustained attention from policymakers, researchers, clinicians, and the public. Radiology is increasingly scrutinized not only for what AI can technically achieve but also for what its adoption reveals about professional roles, responsibilities, and practices. Amid this external scrutiny, radiologists face a pressing intra-disciplinary question: *What will the future of our work look like?*

Most responses to this question fall within 2 dominant frameworks. The first is the job displacement model, which predicts which professions are most at risk of being replaced by AI. This model frequently ranks radiology as especially vulnerable, causing concerns among radiologists and deterring entrants into the profession. The second is the automation-versus-augmentation model, which shifts attention from entire jobs to the level of “tasks associated with jobs.” This model allocates precious resources and attention to some binary divide of probabilities, whether a specific task (contouring, segmentation, patient meeting) may be automated by AI.

Drawing on recent developments in AI and scholarship on technology and organizations, this paper critically examines both frameworks and introduces a third, emerging perspective: **work reconfiguration**. Rather than asking whether AI will replace radiologists or which tasks AI will automate, the reconfiguration perspective focuses on how AI reshapes the *relationships and connections* between tasks, redistributes responsibilities across professional groups (including physician extenders and non-radiologists), and transforms workflows, accountability, and education.

## Why the job- and task-based models fall short

AI’s early success in radiology was driven by its ability to automate narrow image-recognition tasks. High performance in these domains fueled claims that radiologists might soon become obsolete. These claims relied on a reductive logic: if AI can replicate a core task, the profession itself is at risk.

This job-level reasoning obscures the complexity of radiological work. Radiologists do far more than interpret images. They contextualize findings with patient history, manage diagnostic uncertainty, consult with other clinicians, and contribute to clinical decision-making. These interpretive, relational, and judgment-based tasks remain beyond AI’s current capabilities.^1^ Like many professions, radiology cannot be reduced to a single task without distorting what the work entails in real-world conditions.

In response to the limitations of job-level reasoning, a task-based approach has gained prominence in research on technology and employment. From this perspective, jobs are composed of multiple discrete tasks, some of which may be automated by AI while others may be augmented by human-AI collaboration. This nuanced reframing focuses attention on which tasks within each job are most susceptible to “AI automation” or “AI augmentation”—a divide^4^ that increasingly underpins many projections on the future of work.^5^ Cutting-edge research^6^^,^^7^ relies on structured task databases such as O*NET, which catalogs thousands of tasks across hundreds of occupations.^8^ This offers a standardized foundation for researchers and AI developers to understand how automation versus augmentation might differentially affect outcomes such as time savings and efficiency.^7^ For radiologists, O*NET identifies 30 discrete tasks, with narrow, repetitive, and data-rich tasks—such as segmentation or certain administrative tasks—often highlighted as having high automation potential^7^^,^^9^ and suggested to have efficiency gains (eg, time savings).

However, assumptions about AI-driven task-level outcomes in radiology have yet to be validated through robust empirical studies. Even more, AI tools focused on a discrete task may perform very well in isolation, but in real-world clinical settings, the AI’s anticipated efficiency gains may be offset if the AI use disrupts workflows or generates new oversight burdens. Thus, task-level productivity gains—such as time savings—may fail to translate into meaningful improvements at the level of jobs, departments, or patient care.

## Moving beyond the divide: advancing the reconfiguration approach

The task-based model rests on a critical assumption: that professional work can be decomposed into modular, separable units (tasks). In the real world, clinical settings, this assumption often fails. because work is deeply interdependent, embedded in workflows beyond one (medical) specialty, and shaped by organizational routines, regulatory constraints, and professional norms.

In practice, automating a single task may introduce new frictions elsewhere. Evidence from rare longitudinal studies of AI deployment in hospitals^10^^,^^11^ shows that reassigning even a narrowly defined task to be automated by AI—such as outlining organs at risk—can alter accountability structures, coordination patterns, and professional boundaries. Crucially, how AI outputs (eg, the outline of organs-at-risk) are integrated into the surrounding system of work,^11^ generates cascading effects demonstrating that even when AI can effectively automate a discrete, narrow task, the surrounding work is inevitably **reconfigured**.

This paper advances what I term the **reconfiguration approach**—a perspective that views a job not as a bundle of modular, isolatable tasks, but as an integrated system of practice. In this view, tasks are interdependent and shaped by institutional norms (such as regulation and policy), professional judgment, and broader social context. This perspective aligns with foundational work on human–machine reconfigurations,^12^ which emphasizes that technologies transform work by reshaping relationships between people, tools, and organizations.

**Figure 1:**
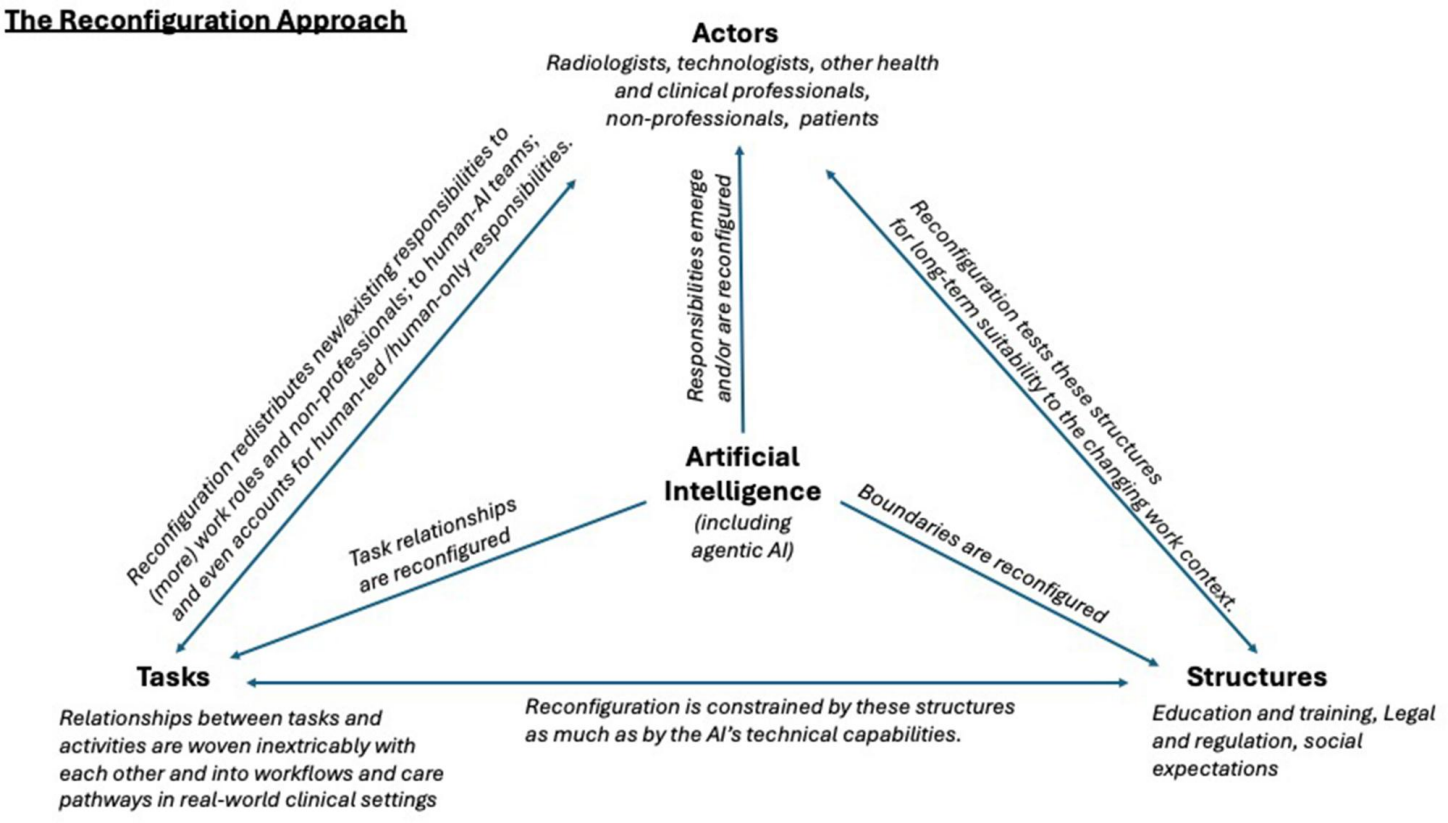
A visual diagram of the reconfiguration approach.

Figure 1 offers an illustration of the reconfiguration approach.

A central dimension of the reconfiguration approach is that any change to individual tasks inevitably affects the wider system of work. Therefore, AI’s impact is not defined solely by whether specific tasks within a job are automated or augmented. Importantly, the reconfiguration perspective acknowledges that some tasks may even remain unchanged—an outcome largely overlooked by the automation-augmentation divide. Rather, the reconfiguration approach highlights that AI’s more consequential effects lie in how it reshapes relationships between tasks, alters clinical decision-making, redistributes responsibility across humans and machines, and transforms accountability structures and clinical workflows across professions and across professionals and their clients (eg, patients).

By extension, a critical dimension of reconfiguration concerns how work redistributes across professional groups. As AI may take on tasks traditionally performed by radiologists—such as measurements, prioritization, or protocoling—responsibilities may shift not only to machines but also to radiographers, technologists, physician extenders, and other non-radiologist clinicians.

Reconfiguration, therefore, is not solely about human–AI interaction but about how AI reshapes the *division of labor* across the imaging ecosystem. This dimension was largely absent from task-based approaches, with limiting implications for our understanding of how AI affects work in practice.

These central dimensions of the reconfiguration approach render it ideally suited for understanding the effects of *agentic AI systems* in real-world work settings. Agentic AI refers to systems that autonomously initiate actions, make context-sensitive decisions across multiple tasks, and even manage workflows across a pathway of care and across multiple medical and non-medical specialties.^13^ In radiology, such agentic AI systems may dynamically manage worklists and orchestrate interactions between imaging, reporting, and downstream clinical processes. Agentic AI cannot be designed or deployed by simply optimizing discrete tasks in isolation, and therefore a task-based lens will not suffice. Without understanding how work is configured in practice—how tasks interrelate, flow, and anchor accountability—agentic AI cannot function safely or effectively. Agentic AI therefore does not merely benefit from a reconfiguration approach; it makes it indispensable.

### Realigning roles, responsibilities, training, and education

As AI-driven reconfiguration of work unfolds, familiar boundaries in radiology are already recognizably shifting. Expanded roles for technologists in AI-assisted workflows may require new competencies and accountability structures. Similarly, as non-radiologist clinicians gain access to AI-generated imaging insights, the boundaries of diagnostic authority may blur. At the same time, new responsibilities are emerging: reviewing AI outputs, managing shared accountability, and navigating medico-legal ambiguities in human–AI collaboration.

These shifts carry important implications for the future workforce, with significant implications for professional identity and training pathways. Radiologists may evolve into workflow supervisors, diagnostic consultants, or integration specialists, overseeing system-level coordination. Reconfiguration therefore requires intentional decisions about how responsibilities are redistributed across professions, and which tasks should remain human-led.

### Boundaries that shape reconfiguration

Reconfiguration does not imply that all aspects of radiological work can—or should—be redistributed to AI or other professionals. Instead, it brings into focus the boundaries that actively shape how reconfiguration can unfold in practice. These boundaries are educational, regulatory, legal, and social, and they define which aspects of work must remain human-led even as others change.

Training represents one such boundary. While AI may reduce repetitive or routine tasks, these activities have traditionally played a critical role in developing clinical judgment. Without sufficient exposure to foundational work, trainees may struggle to acquire the expertise needed to supervise AI systems, interpret uncertainty, and make complex clinical decisions later in their careers. Reconfiguration therefore requires deliberate decisions about which tasks should remain human-led during training, not despite AI, but because of it.

Regulatory and legal frameworks impose further constraints. In most jurisdictions, radiologists remain legally responsible for diagnostic accuracy, report validation, and the interpretation of uncertain findings. These obligations limit how responsibility can be redistributed, regardless of technical capability. As a result, reconfiguration reinforces the need for human–artificial intelligence (HAI) teams where human and non-human agents work together within clearly defined roles and accountability structures.^14^ In radiology, such teams are likely to become the norm, demanding new approaches to education, professional governance, and leadership.

Finally, reconfiguration extends beyond professional and institutional boundaries to patients themselves. As patients gain increasing access to AI tools and health information, perceptions of expertise, authority, and trust may evolve. How radiologists communicate the role of AI, contextualize algorithmic outputs, and maintain human oversight will be central to patient safety and public trust. Importantly, societal expectations continue to favor human authority in clinical decision-making, even when AI performs at a high level. These expectations form a final, powerful boundary shaping how reconfiguration can—and should—proceed.

Interventional radiology (IR) provides a clear illustrative case for these boundaries. The subspecialty of IR combines imaging expertise with technical dexterity, real-time decision-making, and direct patient interaction. While AI may enhance planning, guidance, and procedural safety, it is difficult to envision autonomous systems replacing the embodied, situational judgment required during interventions—and even if AI could, would we want AI to? The boundaries we decide now may shape work reconfiguration for future generations of professionals. Even more, the very choice of framing AI as a tool that reconfigures, rather than replaces, professional work may help attract future radiologists^15^ to the profession, while displacement narratives have instead contributed to professional anxiety.^16^

## Conclusion

The future of radiological work will not be shaped solely by technological capabilities but by how the profession chooses to adapt, govern, and lead. The reconfiguration model offers a more realistic and constructive way to understand this evolution. Radiology is not headed for obsolescence but for transformation.
